# Retrospective Characterization of the 2006–2007 Swine Vesicular Disease Epidemic in Northern Italy by Whole Genome Sequence Analysis

**DOI:** 10.3390/v13071186

**Published:** 2021-06-22

**Authors:** Giulia Pezzoni, Arianna Bregoli, Chiara Chiapponi, Santina Grazioli, Antonello Di Nardo, Emiliana Brocchi

**Affiliations:** 1 Istituto Zooprofilattico Sperimentale della Lombardia e dell’Emilia Romagna, 25124 Brescia, Italy; giulia.pezzoni@izsler.it (G.P.); arianna.bregoli@izsler.it (A.B.); chiara.chiapponi@izsler.it (C.C.); santina.grazioli@izsler.it (S.G.); emiliana.brocchi@gmail.com (E.B.); 2 The Pirbright Institute, Pirbright, Woking, Surrey GU24 0NF, UK

**Keywords:** swine vesicular disease, epidemiological tracing, outbreak investigation, phylodynamics, transmission tree, Italy

## Abstract

Advances in the epidemiological tracing of pathogen transmission have been largely driven by the increasing characterisation of whole-genome sequence data obtained at a finer resolution from infectious disease outbreaks. Dynamic models that integrate genomic and epidemiological data further enhance inference on the evolutionary history and transmission dynamics of epidemic outbreaks by reconstructing the network of ‘who-infected-whom’. Swine Vesicular Disease (SVD) was present in Italy from 1966 until 2015, and since the mid-1990s, it has mainly been circulating within Italy’s central-southern regions with sporadic incursions to the north of the country. However, a recrudescence of SVD in northern Italy was recorded between November 2006 and October 2007, leading to a large-scale epidemic that significantly affected the intensive pig industry of the Lombardy region. In this study, by using whole-genome sequence data in combination with epidemiological information on disease occurrences, we report a retrospective epidemiological investigation of the 2006–2007 SVD epidemic, providing new insights into the transmission dynamics and evolutionary mode of the two phases that characterised the epidemic event. Our analyses support evidence of undetected premises likely missed in the chain of observed infections, of which the role as the link between the two phases is reinforced by the tempo of SVD virus evolution. These silent transmissions, likely resulting from the gradual loss of a clear SVD clinical manifestation linked to sub-clinical infections, may pose a risk of failure in the early detection of new cases. This study emphasises the power of joint inference schemes based on genomic and epidemiological data integration to inform the transmission dynamics of disease epidemics, ultimately aimed at better disease control.

## 1. Introduction

Swine vesicular disease (SVD) is a viral infectious disease in pigs caused by a member of the *Enterovirus* genus (*Enterovirus B* species) of the family *Picornaviridae* [1]. Vesicular lesions are usually found on the coronary bands of infected animals, but vesicles might also appear on their snout, lips, tongue, and teats. Differential laboratory diagnosis with foot-and-mouth disease (FMD) is critical due to the indistinguishable features of the clinical manifestations of the disease. For this reason, SVD was included in the World Organisation for Animal Health (OIE)’s list of notifiable diseases until 2015. However, compared to FMD, SVD is moderately contagious and is characterised by lower morbidity, milder clinical signs, and may progress subclinically in pigs. Pathogenesis varies according to the infecting virus strain, age of the host, route and dose of infection, and the farming conditions [2]. Disease transmission between farms is mainly linked to the movements of infected animals and/or via fomites, despite a limited tendency for the virus to diffuse even at the within-farm level [2,3,4]. The SVD virus (SVDV) is a single-stranded, non-enveloped, positive-sense RNA virus of ~7.4 kb in length. All of the viral lineages belong to a single serotype, which is further characterised by four antigenic/genomic variants [5]. Clinical cases of SVD were initially reported in October 1966 from two farms in the Lombardy region of Italy [1]. The virus was also isolated during late 1971 in Hong Kong [6]. Although the exact geographical origin of the SVDV still remains to be elucidated, its biological origin has been ascribed to co-infection, recombination, and a single anthroponotic transfer event which may have resulted from large viral meningitis epidemics recorded around 1960–1961 [7]. *Enterovirus B* serotype coxsackievirus B5 and A9 were regarded as the most likely viruses involved in this evolutionary process [7]. The SVDV introduction in Europe has allegedly been attributed to the importation of animals and/or animal products from the Far East, however the exact time of the introduction into Europe is uncertain since SVD cases might have been going undetected due to the magnitude of FMD cases reported in the 1960s [7]. Starting from the 1970s, the SVDV has been reported in countries in both Western and Eastern Europe, with outbreaks sequentially caused by the three different antigenic/genomic variants identified after the first one was detected in 1966 [5]. Since the early 1990sand after the latest outbreaks were reported in the Netherlands, Belgium, and Spain, SVD has been eliminated from most of the European territories, with the exception of Portugal and Italy. From then on, sporadic recurrences of the disease in Portugal were evidenced in 2003 and 2007 [8]. In Italy, the fourth SVDV antigenic variant [5] has been circulating for more than 20 years, mainly in Italy’s southern regions with sporadic incursions in the north of the country. During the last 20-year period, two different lineages have been detected in Italy: one evolved exclusively in Italy from viruses first introduced in 1992, and the second one was detected in2004 and is closely related to the viruses circulating in Portugal during 2003, 2004, and 2007 [8,9].

**Figure 1:**
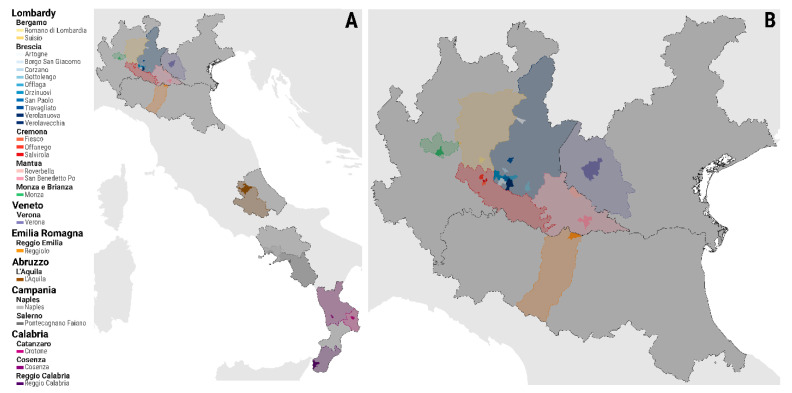
Geographical distribution of the Italian municipalities affected by the 2006–2007 SVD epidemic investigated in this study. Full geographical extent of the epidemic event including the southern Italian cluster (**A**) and the northern epidemic cluster only (**B**). Geographical locations are presented as region (first admin-level), province (second admin-level), and municipalities (third admin-level).

The last major epidemic of SVD occurring in Northern Italy was reported in 2006, causing a total of 53 outbreaks with the culling of ~150,000 animals and an estimated financial impact of ~EUR 30 million [3]. On the 2nd of October 2006, pigs were tested SVD seropositive at a slaughterhouse in the Italian province of Bergamo (within the Lombardy region) after 4 years of SVD being absent from northern Italy [3]. During the 11 months of the epidemic course recorded from November 2006 to October 2007, SVD spread widely in a densely populated pig area of Lombardy (Figure 1). The epidemic event was characterised by two subsequent phases, with the recrudescence of infections detected at a 4 month from the last SVD case identified during the initial phase (Supplementary Figure S1). During the same time period, SVD outbreaks were also reported in both central and southern Italy (Supplementary Figure S1), but these were not found to be linked with the Lombardy cluster, according to field epidemiological tracing.

In this study, 37 whole-genome sequences (WGS) generated from SVD isolates collected from farms affected during the entire timeframe of the epidemic and 6 further WGSs of contemporary viruses isolated from the SVD outbreaks reported in southern Italy (Figure 1A) have been used to retrospectively retrace the transmission history of the epidemic by co-analysing the epidemiological and genetic data. We report a detailed reconstruction of the epidemic event, describing the space and rate of its epidemiological and evolutionary scales and providing evidence for undetected silent/sub-clinical virus transmissions. Knowledge of the risk factors for infectivity and transmission gained by a thorough understanding of the dynamics of infections can be translated into more effective interventions for better disease control.

## 2. Materials and Methods

### 2.1. Epidemiological Field Investigation and Data Collection

During the epidemic and as prescribed by the Italian national surveillance plan for SVD [4,10], a farm was defined as having a suspected case of SVD when: (i) one single pig showed clinical signs; or (ii) one single pig tested seropositive; or (iii) an epidemiological link with an officially confirmed SVD infected farm was established. Following the reporting of a suspected case of SVD, each farm was subjected to a detailed clinical and epidemiological veterinary investigation in order to establish (i) the possible time of infection, (ii) the likely origin of infection and the links between other infected (or suspected of being infected) farms, and (iii) the most likely conveyor of infection. Epidemiological data collected during field investigations and analysed in this study (Table 1) were retrieved from the original paper-based forms and stored in an electronic database.

### 2.2. Clinical Samples

Sampling procedures were carried out along with epidemiological investigations and were aimed at collecting epithelium, vesicular fluid and faecal samples for virological testing, and blood samples for serological testing. Virological tests consisted of a RT-PCR assay and virus isolation on the IBRS2 cell line [11] and serological assays included a specific competitive ELISA and an indirect ELISA for the identification of the isotype of the antibody present in the positive sera in order to evaluate the time of the exposure to the infection [11]. A total of 43 SVDV isolates derived from epithelium or faecal samples were analysed in this study, 37 of which were collected from pig farms in Lombardy between November 2006 and October 2007 (Table 1).

### 2.3. RNA Isolation, PCR Amplification and Sequencing

The RNA amplification method applied to prepare the samples for whole-genome sequencing consisted of a classical RT-PCR producing two overlapping amplicons performed on RNA extracted from viruses isolated in IBRS-2 cell culture [11]. First, the RNA extraction was performed from 140 μL of infected cell culture supernatant with a QIAamp viral RNA Mini Kit (Qiagen^®^ Ltd., Manchester, UK) according to the manufacturer’s instructions. Five microliters of RNA were amplified by RT-PCR, performed using the SuperScript™ III One-Step RT-PCR System with Platinum™ *Taq* High Fidelity DNA Polymerase (Invitrogen, Thermo Fisher Scientific, Loughborough, UK). Two copies of the primers were used: FOR1VP1 (5′TCTCAGTKAGGATGCTCAAGGATAC3′) along with REVPOLIA (5′TTTTTTTTTTTTTTTTYYYYNCCGCACC3′) to amplify a portion of 5012 pb and 5′UTR FOR SH (5′TGTGGGTTGTTCCCACCCAC3′) and REV2VP1 (5′AGTTGCCGACRTAK ACAGC3′) to amplify a portion of 3315 bp. The forward primers were used at the final concentration of 0.2 μM while the reverse primers were used at at 0.5 μM. The thermal profile was as follow: RT at 55 °C for 30 min, pre-denaturation at 94 °C for 2 min, and 40 cycles of denaturation at 94 °C for 15 s, annealing at 60 °C for 30 s, extension at 68 °C for 5 min, and final extension at 68 °C for 5 min. The amplified overlapped regions that covered almost the entire genome were analyzed in a 1% agarose gel stained with EuroSafe Fluorescent (EuroClone, Pero (MI), Italy) and gel-purified using the NucleoSpin^®^ Gel and PCR Clean-up kit (MACHEREY-NAGEL GmbH & Co. KG, Germany). The DNA was eluted with 25 μL of buffer NE (from the kit), quantified with the NanoQuant Plate (Tecan Group Ltd., Männedorf, Switzerland) spectrophotometer, and submitted to the MiSeq (Illumina Inc., San Diego, CA, USA) platform for sequencing. The Nextera XT DNA Library Prep Kit (Illumina Inc., USA) was used to generate multiplexed paired-end sequencing libraries, according to the manufacturer’s instructions. De novo genome assembly was performed using Lasergene SeqMan NGen^®^ and SeqMan Pro 12 (DNASTAR Inc., Madison, WI, USA).

The length of the 43 WGSs generated ranged from 7347 bp to 7423 bp, including the ORF sequence encoding the viral polyprotein. To account for difficulties in resolving the full-consensus sequence for some of the isolates, all of the WGSs analysed were trimmed to a length of 7333 bp, including regions at the 5′ UTR (717 bp in length) and the 3′ UTR (58 bp in length), with the ORF resulting in a length of 6558 bp. Twenty-one SVD isolates showed a six-nucleotide (nt) deletion in the 5′ UTR region. The coverage for all of the generated WGSs was estimated to have an average value of 9.8 × 10^2^ and ranged between 3.0 × 10^3^ and 1.1 × 10^1^. Consensus sequences were aligned using MAFFT 7.475 [12]. All of the strains were characterised as belonging to sub-lineage 1 of the fourth SVDV antigenic/genomic variant; this sub-lineage comprises of viruses that evolved in Italy after its original introduction in 1992 [9]. 

### 2.4. Phylogenetic Inference and Phylogeography Reconstruction

Molecular evolution was modelled according to the GTR+Γ4 substitution model [13], following establishment of the best-fit model of nt substitution using jModelTest 2.1.10 [14,15]. Evolutionary rates were allowed to vary across tree branches according to a log-normal relaxed molecular clock [16], with the non-parametric skyline demographic model set as the prior density on the trees [17]. For the overall evolutionary rate, ‘prior’ was defined as an exponential distribution with mean equal to 1, whilst all other priors used to infer the phylogenetic tree were left at their default values, as assigned in BEAUti 10.1.5 [18]. To reconstruct the history of the movements of the SVDV variants across geographical locations (i.e., provinces and regions), a discrete-trait phylogeography approach was used, employing an asymmetric (non-reversible) continuous-time Markov chain (CTMC) model along with a Bayesian Stochastic Search Variable Selection (BSSVS) procedure [19]. Either the first or second administrative divisions of Italy (i.e., regions and provinces, respectively) were defined and assigned to each of the genetic sequences as a discrete trait location. In addition, a Markov Jumps (MJ) procedure was used to reconstruct a stochastic realisation of the between-location diffusion process, described by the median numbers of transitions between each pair of geographical locations [20]. A joint posterior estimate of the genetic and phylogeographic models was obtained by running a Markov Chain Monte Carlo (MCMC) chain of 200 million states in BEAST 1.10.5 [18], sampling every 20,000 states. The mixing and convergence of the MCMC chain was assessed using Tracer 1.7.1 [21], ensuring sufficient sampling was achieved (ESS ≥ 200). The initial sample of 1001 trees were removed as burn-in, and the remaining 9000 were used to infer the final Maximum Clade Credibility (MCC) tree. Maps were plotted in R 4.0.4 [22] along with phylogenies using the *ggtree* 2.5.1 package [23].

### 2.5. Transmission Tree Reconstruction

Inference of the outbreak transmission tree was performed using the Bayesian model framework implemented in the *outbreaker2* 1.1.2 package for R [24]. The generation time (w) of SVD was described as having a gamma probability distribution of the form w~Γ(κ,θ), with shape and scale parameters set to a value of 1.64 and 14.19, respectively, which defines a time extent with mean of 23.3 days and variance 329.1 [25]. The resulting distribution was then rescaled to sum to 1. Data were analysed using the parallelised implementation of the model with eight independent MCMC chains each run for 200,000 iterations with a thinning frequency of 1/200. The results were based on the posteriors samples merged across runs after discarding a burn-in of 10% of the chain and examining for convergence. The reconstructed transmission tree has been edited and plotted using Visone 2.19 [26].

### 2.6. Testing for Selection in the SVDV Genome

Potential selective pressures acting on the SVDV protein coding genes during the 2006–2007 SVD epidemic were investigated by performing two analyses in HyPhy 2.5.31 [27]: (i) the differences in selective pressure between the two phases of the epidemic were characterised using Contrast-FEL (Fixed Effects Likelihood) [28], which was also used to estimate site-specific synonymous and non-synonymous substitution rates; (ii) residues subject to directional selection along the reconstructed phylogeny were mapped using FADE (FUBAR Approach to Directional Evolution) [27].

## 3. Results

### 3.1. Phylogenetic and Phylogeography Analyses

**Figure 2:**
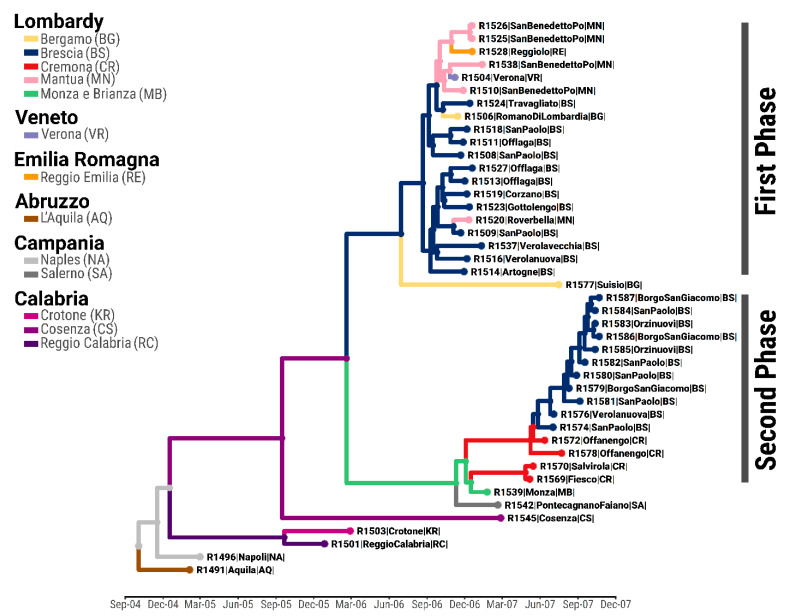
Phylogeographic history of the 2006–2007 SVD epidemic in northern Italy reconstructed from the 43 full-length SVDV genome sequences. Branches and tips are coloured according to the posterior geographical ancestry resulting from the Bayesian discrete asymmetric trait analysis with the province inputted as a discrete trait.

The time-resolved phylogeny reconstructed from the 43 SVDV WGSs designated the phylogenetic cluster linked to the SVD epidemic in northern Italy as characterised by two distinct clades diverging from a single ancestral virus allegedly circulating in southern Italy (Calabria province, Posterior Probability (PP) = 0.61) at the end of February 2006 (95% Bayesian Credible Interval (BCI) November 2005 to June 2006) (Figure 2; Supplementary Figure S1). In addition, inferences based on the Bayesian phylogeography analyses at the province level revealed two different geographic sources of infections for the first and second phases of the epidemic event.

### 3.2. Ancestry of the First Epidemic Phase

The initial introduction of the SVDV in northern Italy was estimated to be at the end of June 2006 (95% BCI April 2006 to August 2006) (Supplementary Figure S2), assigning the province of Brescia as the likely source of virus movements during the initial epidemic phase with high probability (PP = 0.84). Several geographic transitions were reconstructed between June and November 2006 within Brescia province (mean PP of 0.98 ± 0.04; median MJ count of 26), with viruses further moving from there into the province of Mantua on two occasions (time of the most recent common ancestor (tMRCA) is 23rd September 2006 and 3rd November 2006, respectively) and moving into the province of Bergamo once (tMRCA 9th October 2006) (Figure 2; Supplementary Figure S2). The farm in Emilia-Romagna region (south of Lombardy) that reported being infected in December 2006 was found to be linked with a phylogenetic cluster of SVDVs that were isolated from different infected premises located in Mantua province (tMRCA 29th October 2006; PP = 0.81).

### 3.3. Ancestry of the Second Epidemic Phase

**Figure 3:**
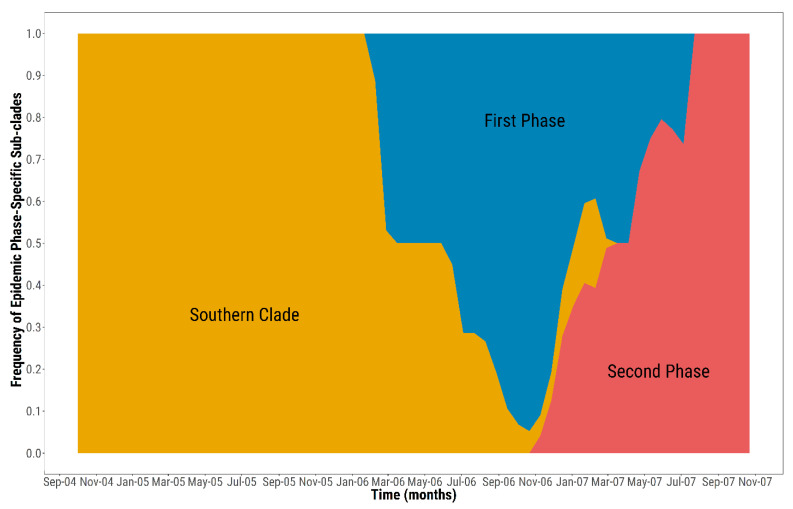
Inferred frequency of epidemic phase specific SVD lineages characterising the 2006–2007 SVD epidemic in northern Italy. The *y*-axis shows the proportion of SVD sub-clades contributing temporally to the epidemic ancestry makeup, while the *x*-axis represents the root-to-tip time extent.

By quantifying the average ancestry of the SVDV variants that characterised each of the epidemic phases (Figure 3), viruses linked with the second wave of SVD cases were found to have been evolving by the end of 2006, thus earlier than reported, progressively increasing in frequency, and seeding half of the infections by April 2007.

Genealogically clustering within the second epidemic phase, these SVDV isolates were reconstructed to coalesce to a common ancestor (tMRCA December 2006, 95% BCI October 2006 to January 2007) shared with the last SVD case reported in the province of Monza at the end of the first epidemic phase, although geographic transitions were assigned with low posterior support (average PP of 0.43 ± 0.07) (Figure 2; Supplementary Figure S2). Sequence similarity analyses further showed the virus genome isolated in January 2007 from the infected farm in Monza as significantly homologous to the second wave of infections reported 4 months later in May 2007 (2-sided Kolmogorov–Smirnov test, D = 0.94, p = 0.000). During this second epidemic phase, virus movements were identified from Monza to Cremona province (tMRCA April 2007; PP = 0.46), where the SVD cases were initially reported, and from there to Brescia (tMRCA May 2007; PP = 0.98), where the SVDV infection spread sequentially in time across different municipalities (average PP of 0.98 ± 0.06; median MJ count of 20) (Figure 2; Supplementary Figure S2). The line of descent of the second wave of SVDV infections was also connected to a virus isolated from an infected premise reported in southern Italy (Campania region) in February 2006, despite low statistical confidence in the reconstructed geographic transition (PP = 0.35).

### 3.4. Transmission Tree Structure and Epidemiological Features of the Epidemic

**Figure 4:**
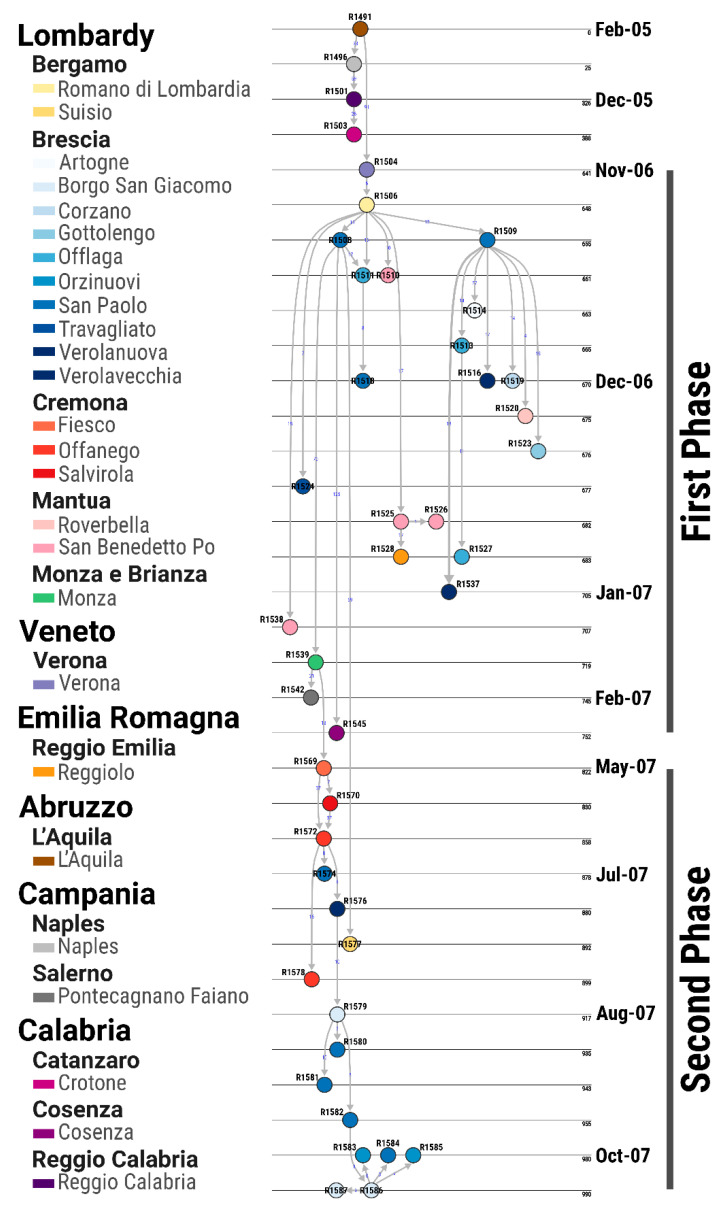
Transmission tree of the 2006–2007 SVD epidemic in northern Italy mapped in time. Nodes are coloured according to the municipality of the farm location. Blue numbers reported close to the arrows represent nt changes estimated between the SVDV genome sequences isolated from each of the linked farms.

**Figure 5:**
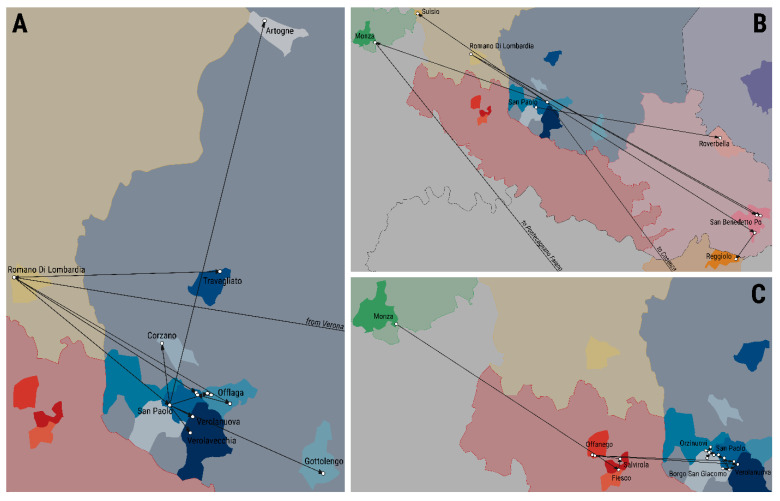
Spatial migration history of SVD infections recorded during the 2006–2007 epidemic across the affected geographical area of northern Italy. Initial spread of SVD infections within the province of Brescia (**A**) and subsequent geographic transitions (**B**) during the first epidemic phase; virus movements reconstructed for the second epidemic phase (**C**).

Results of the inferred transmission tree provided a high posterior support (>0.9) for 92.8% (39/42) of the reconstructed links (Table 1; Figure 4). The main initial hub of the SVDV transmissions in northern Italy was identified in a livestock dealer’s premises (R1504) located in the province of Verona (Veneto region), which was described as central in the early spread of infections that characterised the initial cluster of outbreaks recorded in the municipality of San Paolo (Brescia province) (Figure 4 and Figure 5). This first phase of the epidemic was characterised by short chains of transmissions of which only 11/15 were first generation of infections, thus strengthening the role of infecting hubs (i.e., of R1508 and R1509 that were reported in San Paolo) in the initial network of transmissions.

Multiple infected premises that were later reported in Mantua were also found to be directly linked to the index case (i.e., R1504/R1506), with the exclusion of the farm discovered in Roverbella that was reconstructed as being directly connected to the disease cluster of San Paolo (Brescia). The epidemiological link between the two phases of the epidemic was identified in the infected premises of Monza (R1539), which resulted an intermediate phase in the time and evolutionary space of the epidemic. Despite having been reported in January 2007 during the first epidemic phase, this farm was found to be evolutionarily distant by one generation of infections from the cluster of outbreaks reported in Cremona (R1569, R1570 and R1572) during the onset of the second phase (Figure 4). The movement of the viruses were then sequentially reconstructed from Cremona to Brescia, and within Brescia province between the municipalities of Borgo San Giacomo and San Paolo (Figure 4 and Figure 5). Thus, in contrast to the initial epidemic phase where multiple transmissions were seeded from infecting farm hubs, in this second phase, individual farms were infected at any one point in time, with a rapid turnover of transmissions through time. Interestingly, the only infected premises discovered in Bergamo province (Suisio, R1577) in late July 2007 was linked to the initial cluster of infections reported early in San Paolo during the first phase of the epidemic.

The posterior proportion of farms sampled across the entire epidemic was estimated at an average value of 0.81 ± 0.07, which indicated likely missing links along the chain of SVDV transmissions.

### 3.5. Case-Reproduction Ratio and Spatial Connectivity 

**Figure 6:**
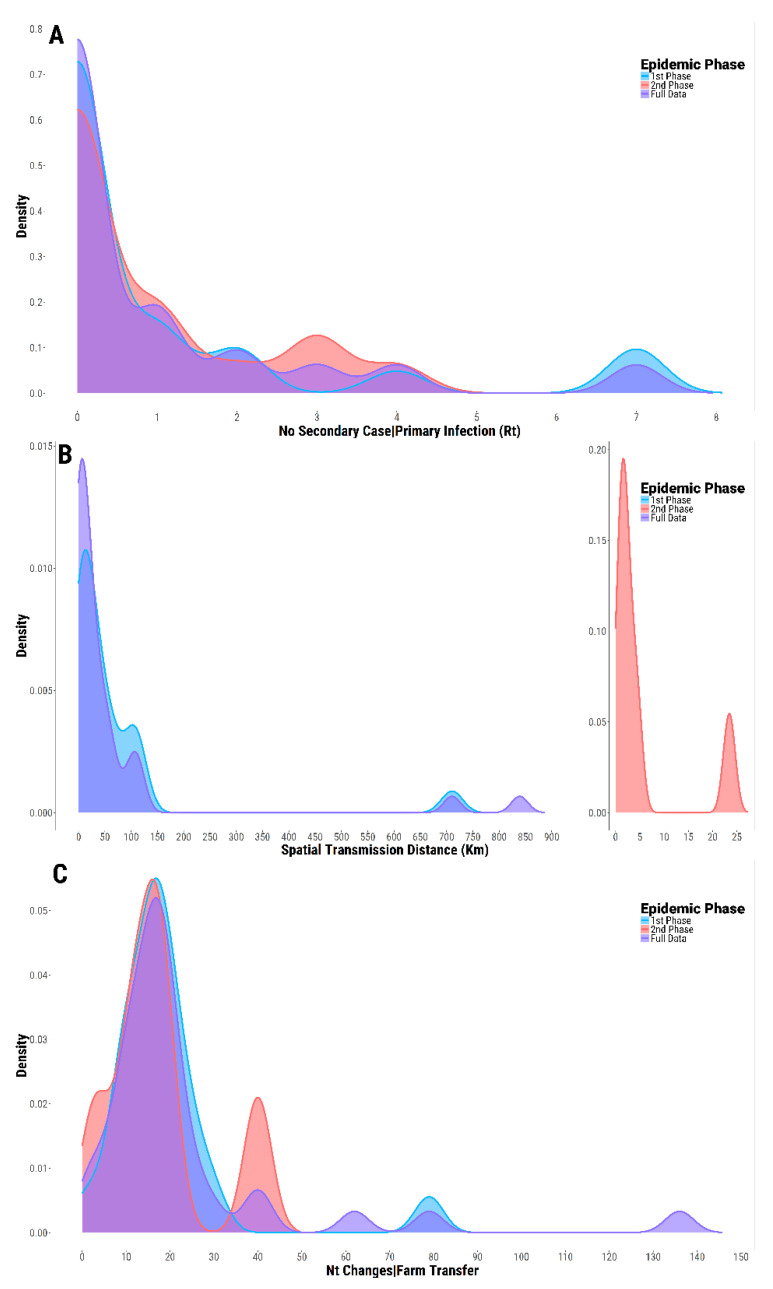
Epidemiological parameters characterising the 2006–2007 SVD epidemic in northern Italy. Kernel density distributions estimated for: the reproduction number $R_{t}$ (**A**); the spatial transmission distances between linked farms (**B**); the nt changes acquired per farm transfer (**C**), respectively, derived for the full timeframe of the epidemic (violet), the first (blue), and second (red) epidemic phases.

The average number of secondary farms arising from a single infected premise ($R_{t}$) estimated for the full timeframe of the epidemic (but excluding the initial outbreaks reported from Southern Italy) resulted in a value of 1.0 (95% PI 0–6.8). A slightly higher $R_{t}$ value was described for the first epidemic phase (1.09, 95% PI 0–7.0), as opposed to the second phase (0.88; 95% PI 0–3.6) (Figure 6A).

The spatial transmission distance between the linked premises was estimated at an average value of 67.7 km (95% PI 0.4–723.3) for the entire epidemic (Figure 6B), with only 24.3% (9/37) located within the protection zone of 3 km. The second phase of the epidemic was yet characterised by a more localised spread with a short-distance transmission of infections (an average distance of 5.7 km, 95% PI 0.6–23.6), 58.3% (7/12) of which were geographically contained within the extent of the 3 km protection zone.

The large mean transmission distance evidenced for the first epidemic phase may be explained by the long distance connections allegedly reconstructed between SVD outbreaks reported in February 2007 in southern Italy and the Lombardy cluster: (i) the infected premises reported in Calabria (R1545) was found to be linked with the initial cluster of infected farms discovered in late 2006 through field investigation in San Paolo (linear distance of 839.1 km), although the analysis provided moderate posterior support (0.75) for this transmission link; (ii) the infected premises located in Campania (R1542) established to be connected to the last reported case of SVD in Monza (R1539) (linear distance of 710.4 km).

### 3.6. Evolutionary Profile of SVDV Transmissions

**Figure 7:**
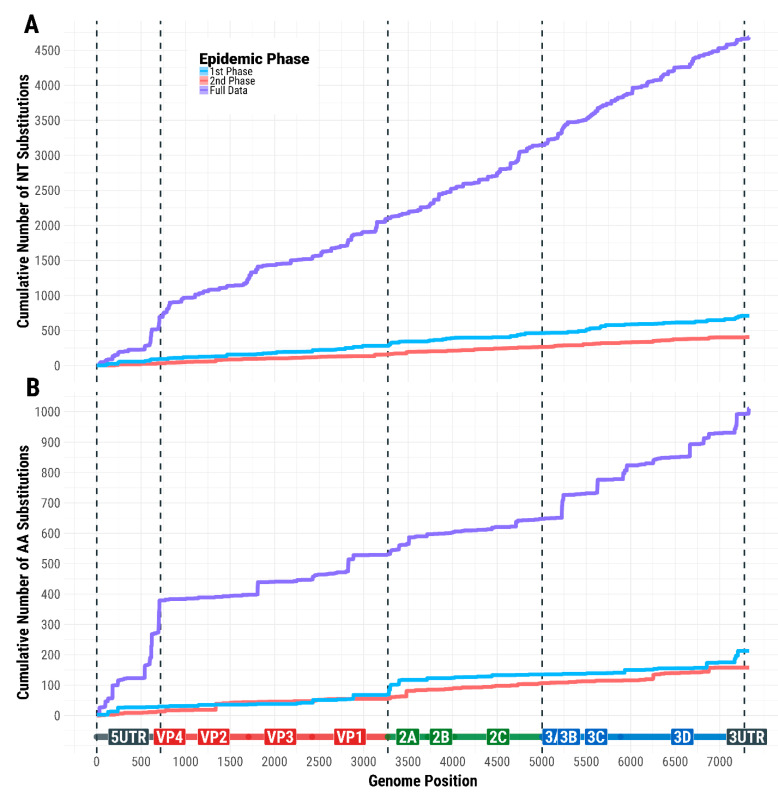
Nucleotide and amino acid substitutions occurring along the SVDV genome during the 2006–2007 SVD epidemic in Italy. (**A**) Distribution of the cumulative number of nt substitutions across the different genomic regions of SVDV; (**B**) distribution of the cumulative number of amino acid substitutions across the different genomic regions of SVDV.

Within the timeframe of the epidemic, the SVDV evolved at a rate of 1.73 × 10^−5^ nt/site/day (95% BCI 1.34 × 10^−5^–2.14 × 10^−5^), showing a very strong signal of a linear evolutionary trend ($R^{2}$ = 0.96). Nucleotide substitutions were observed throughout the SVDV genome (Figure 7), estimating a total number of 551 segregating sites from the full set of 43 SVDV sequences, where only 208 and 118 polymorphisms were found in the sequences generated from samples collected during the first (*n* = 21) and second (*n* = 17) epidemic phases, respectively. The distribution of nt changes across the SVD genome was found to be different between the first and second epidemic phases (z = 7.922, p < 0.001), with a higher frequency of nt changes accruing within the non-structural regions (P2 and P3 proteins) during the first epidemic phase.

Nonsynonymous/synonymous rate ratios were estimated to be higher during both epidemic phases than those characterising the pre-epidemic evolutionary background (ω = 0.09, 0.147, and 0.171 for the pre-epidemic, first, and second epidemic phases, respectively). Analyses of the evolutionary selective pressure on the SVDV genome identified a total of 4 amino acid residues evolving differently between the viruses causing the 2006–2007 epidemic in northern Italy and those concurrently circulating within southern Italy (Table 2). Positive diversifying selection was reported in two of these residues (residues 999 and 2054; p < 0.05 for ω=β/α>1), which were located in different non-structural regions of the SVDV genome (2A and 3D, respectively). These residues were also found to have evolved differently between the first and second phases of the epidemic.

Further analyses detected a total of three amino acid residues that were directionally evolving under selection, with two of these sites inferred for SVDV nonstructural regions (Table 3). The residue located at position 2054 (3D region) was also found to be under diversifying selection and evolutionarily characterising the first epidemic phase and subject to strong bias towards alanine (B = 40.77).

The number of nt changes acquired per farm transfer was estimated to have an average value of 22.7 (95% PI 1.9–84.7), with no statistical differences observed between the estimates obtained from each epidemic phase (z = 0.094, *p* = 0.925) (first phase 18.9 (95% PI 4.1–53.3); second phase 17.2 (95% PI 2.5–40.0)) (Figure 6C). However, the average number of substitutions accumulated between epidemic phases (84.7, 95% PI 59.5–122.2) was found to be higher than that observed within each epidemic phase (F = 86.47, p < 0.001). Looking in more detail at this unexpectedly high number of mutations evidenced between the two epidemic phases and considering the generation time distribution w inputted for the analysis, the number of consecutive generations of outbreaks that may have been gone undetected (and thus undisclosed) was estimated to have an average value of 3.9 (95% PI 2.7–5.6). There were 3.2 and 2.7 generations of unidentified outbreaks that were also estimated between the San Paolo hub of the first epidemic phase and the two evolutionary outliers that were clustering either in time or phylogenetically within the first epidemic phase, Monza (R1539) and Suisio (R1577), respectively.

## 4. Discussion

A recrudescence of SVD was recorded between 2006 and 2007 in northen Italy during which outbreaks were identified in both the Lombardy and Veneto regions, with a single infection spillover reported in Emilia Romagna that was quickly controlled and extinguished. Despite the cases in Veneto being promptly resolved, in Lombardy, SVD infections continued over two epidemic phases that lasted for one year, affecting a large number of pigs in the most economically important intensive farming area in Italy.

Over the epidemic timeframe, the movements of the pigs and the spatial proximity between farms were assessed as primary routes of transmission that most likely contributed to the spread of the disease. Local transmissions over short distances were mostly reported during the second phase, with infections largely clustering in Brescia province. It has previously been shown how this transmission pattern might be the result of the high density of pig farms present in the area [3,29]. However, when excluding the onset of long-distance transmissions resulting from the infection hub in Verona, a similar epidemiological scenario can also be described for the first phase. The high stability of the SVDV in the environment outside the host creates indirect routes of transmission (i.e., the fomites or movements of infected vehicles and personnel) which likely allow for virus dispersal between-farms [25].

During the 2006–2007 SVD epidemic, only 3 out of the 37 affected farms were detected based on clinical signs; in the remaining 50 affected farms, SVDV infections were identified by routine surveillance activities based on laboratory testing and/or through contact tracing. During close veterinary inspections carried out on these farms, clinical signs were only observed in approximately 50% of these cases. A gradual loss of a typical clinical manifestation of SVD has been more frequently reported in the field [3]. This change in the clinical features of the disease, further linked with an increasing evidence of infections characterised by a sub-clinical course, triggers the potential for virus transmissions going undetected. In this epidemiological scenario, the presence of sub-clinically infected pigs may pose a likely risk of the potential failure of surveillance and eradication activities, missing the early detection of cases.

Epidemiological tracing of SVD cases along with serological surveillance activities conducted during the epidemic provided evidence for the presence of SVD in northern Italy well-before the detection of the first case, which was identified in a slaughterhouse in Bergamo province. On these premises, pigs testing positive for IgM were found during active surveillance investigations conducted in compliance with the national surveillance and eradication programme [3]. The Bergamo abattoir, which processed animals from various sources, traded pigs in June 2006 from a farm in the southern Italian region of Campania, where IgG seropositivity was also reported. IgG antibodies are detected in blood samples of infected pigs starting from the 14th day of post infection and may last beyond 4 months [11]. Using the WGS data for timing the MRCA of the northern Italy epidemic, our results suggest the likely circulation of SVDV within the Lombardy region since late June 2006 (thus predating the start of the epidemic event by 4 months). These findings clearly substantiate the hypotheses that: (i) the SVDV variant that started the epidemic event might have been circulating sub-clinically (or at a very low infectivity level) in a single or multiple undetected farms before the initial cases were identified in the framework of regular sampling and testing activities prescribed by the national surveillance programme; and (ii) the initial introduction of the virus from southern Italy chronologically preceded the epidemic event by at least 4 months. It is not entirely clear yet how the sub-clinical infections affected the evolutionary dynamics of the SVDV variant that shaped the epidemic. 

The analysis of the transmission tree further hints to the likely role of ‘missed cases’ and of virus evolution within—and between—host(s) in the spread of SVDV in Lombardy. Indeed, the reconstructed network of infections suggests that the farm reported in Monza (R1539) during the first epidemic phase would in fact be part of the second phase, and that it was likely infected by some unknown premises (or from a chain of unidentified transmission events) linked with the initial cluster of cases reported in Brescia (San Paolo). Interestingly, farms that have been reported during the second epidemic phase share mutations with the SVDV isolate characterised from the farm in Monza, which are not found on the other premises. This may be deemed surprising, as the farm in Monza was reported within the timeframe of the first epidemic phase. In a hypothetical epidemiological scenario, the virus may have fixed the common substitutions while replicating on one (or more) unsampled farm(s), which would represent the missing nodes in the epidemic network linking the two phases. Indeed, while the collection dates of some of the contemporaneous SVDV isolates reported from the provinces of Brescia and Mantua are relatively close to the one recovered from Monza, viral replications over the number of infections consistent with the generation time of SVD may have allowed this transmission to occur. The probability distribution of the generation time for SVD would thus account for at least three generations of infections likely missed within the chain of observed transmissions.

It is well established that evolutionary forces acting at different stages of infection (i.e., at both the intra- and inter- host levels) affect the maintenance of genetic variation in a virus population and the frequency of mixed-strain infections [30]. Indeed, two important observations can be made along those lines from the topology of the reconstructed phylogeny: (i) the SVDV variant introduced from southern Italy that caused the epidemic in Lombardy evolved in two distinct variants that characterised each of the epidemic phases; (ii) both SVDV variants existed and were likely co-circulating at the same time in Lombardy, but their frequencies varied with time. The evolutionary rate and mode of the SVDV variant circulating during the second phase, which indicates the rapid accumulation of mutations and fitness loss, may indeed suggest earlier inter-host transmission bottleneck events that facilitated the fixation of polymorphisms and led to the rise of two SVDV variants. Nevertheless, the size of the bottleneck at the between-host level drives the likelihood of evolutionary emergence of the minority variant, and whether transmissions of this variant occur rapidly or gradually along the chain of infections [31], with tight bottlenecks resulting in higher mutations observed at the consensus sequence. Virus migration from a less intensive farming system to the relatively higher farm density present in the area affected by the second epidemic phase could have also potentially contributed to the increasing higher proportion of infections started by the second minority variant. It is, therefore, likely that the two SVDV variants that characterised each epidemic phase and identified by polymorphisms of mutations fixed at the consensus level may have resulted from a number of factors. These would include the variation in the degree of bottleneck on the transmitted virus population by different transmission routes and number of virus replication cycles that may have occurred either during- or post- transmission in sub-clinical hosts. This evolutionary scenario is also supported by what has been evidenced through the analyses of the selective pressure acting on the SVDV protein coding genes during the 2006–2007 SVD epidemic, which would likely be indicative of a target residue frequency increase and the maintenance of minority variants that might have contributed to the conditions leading to the different evolutionary signatures characterising the two epidemic phases.

However, it might be also worth considering that alternative explanations, such as the introduction of two variants of the same virus lineage from southern Italy (one of which is divergent by mutations accumulated from viral replications in unknown locations), cannot be also ruled out. For this latter case, analyses of supplemental WGSs generated from contemporary SVD cases reported in southern Italy may help to confirm if a different ancestry exists for the second epidemic clade.

Despite epidemiological tracing activities carried out at the time of the epidemic being able to determine a clear picture of the event, some of the observed epidemiological features of the epidemic were left unexplained. Field information from outbreaks is not always collected at this level of detail; hence, it is not entirely adequate for reconstructing the complete history of virus transmissions between farms. Thus, investigating evolutionary history and predicting the origins of viruses by studying the viral genomics of infectious diseases offer very detailed epidemiological information. By using a dynamic model that integrates genetic and epidemiological data, we were able to correctly resolve 96% and 100% of the transmission scenarios that were established at the time of the epidemic by field epidemiological tracing investigations, respectively, during the first and second epidemic phase. In addition, further computational analyses from the obtained results yield new insights on the evolutionary dynamics of the epidemic, helping to shed light on how evolutionary forces may have shaped the time course of the epidemic event and the potential role of sub-clinical hosts associated with infectiousness and transmission.

## Figures and Tables

**Table 1:** Epidemiological data extracted for each of the SVDV isolates analysed in this study and collected during veterinary field investigations from each of the farms affected by the 2006–2007 SVD epidemic.

Sequence ID	Municipality (Province)	Region	Sampling Date	Source ID	Material Samples	GenBank Accession No	SRA Accession No
R1491	Villa Sant’Angelo (AQ)	Abruzzo	2 April 2005		Faeces	MW984391	SAMN19667915
R1496	Torre del Greco (NA)	Campania	1 March 2005		Faeces	MW984392	SAMN19667916
R1501	Locri (RC)	Calabria	27 December 2005		Faeces	MW984393	SAMN19667917
R1503	Casabona (KR)	Calabria	27 February 2006		Faeces	MW984394	SAMN19667918
R1504	Verona (VR)	Veneto	7 November 2006	*	Faeces	MW984370	SAMN19667894
R1506	Romano Di Lombardia (BG)	Lombardy	14 November 2006	R1504	Faeces	MW984371	SAMN19667895
R1508	San Paolo (BS)	Lombardy	21 November 2006	R1504	Faeces	MW984372	SAMN19667896
R1509	San Paolo (BS)	Lombardy	21 November 2006	R1504	Faeces	MW984373	SAMN19667897
R1510	San Benedetto Po (MN)	Lombardy	27 November 2006	R1504	Epithelium	MW984374	SAMN19667898
R1511	Offlaga (BS)	Lombardy	27 November 2006	R1508	Epithelium	MW984375	SAMN19667899
R1513	Offlaga (BS)	Lombardy	1 December 2006	R1508	Epithelium	MW984376	SAMN19667900
R1514	Artogne (BS)	Lombardy	29 November 2006	R1508	Faeces	MW984377	SAMN19667901
R1516	Verolanuova (BS)	Lombardy	6 December 2006	R1513	Epithelium	MW984378	SAMN19667902
R1518	San Paolo (BS)	Lombardy	6 December 2006	R1508/R1509	Faeces	MW984379	SAMN19667903
R1519	Corzano (BS)	Lombardy	6 December 2006	R1513	Faeces	MW984380	SAMN19667904
R1520	Roverbella (MN)	Lombardy	11 December 2006		Faeces	MW984381	SAMN19667905
R1523	Gottolengo (BS)	Lombardy	12 December 2006	R1513	Faeces	MW984382	SAMN19667906
R1524	Travagliato (BS)	Lombardy	13 December 2006	*	Faeces	MW984383	SAMN19667907
R1525	San Benedetto Po (MN)	Lombardy	18 December 2006	*	Epithelium	MW984384	SAMN19667908
R1526	San Benedetto Po (MN)	Lombardy	18 December 2006	*	Epithelium	MW984385	SAMN19667909
R1527	Offlaga (BS)	Lombardy	19 December 2006	R1513	Epithelium	MW984386	SAMN19667910
R1528	Reggiolo (RE)	Emilia Romagna	19 December 2006	*	Faeces	MW984387	SAMN19667911
R1537	Verolavecchia (BS)	Lombardy	10 January 2007	R1518	Epithelium	MW984388	SAMN19667912
R1538	San Benedetto Po (MN)	Lombardy	12 January 2007	R1510	Epithelium	MW984389	SAMN19667913
R1539	Monza (MB)	Lombardy	24 January 2007	*	Faeces	MW984395	SAMN19667919
R1542	Pontecagnano Faiano (SA)	Campania	19 February 2007		Faeces	MW984396	SAMN19667920
R1545	Cosenza (CS)	Calabria	26 February 2007		Faeces	MW984397	SAMN19667921
R1569	Fiesco (CR)	Lombardy	7 May 2007		Epithelium	MW984398	SAMN19667922
R1570	Salvirola (CR)	Lombardy	15 May 2007	R1569	Epithelium	MW984399	SAMN19667923
R1572	Offanengo (CR)	Lombardy	12 June 2007	*	Epithelium	MW984400	SAMN19667924
R1574	San Paolo (BS)	Lombardy	2 July 2007	*	Epithelium	MW984401	SAMN19667925
R1576	Verolanuova (BS)	Lombardy	4 July 2007	R1574	Epithelium	MW984402	SAMN19667926
R1577	Suisio (BG)	Lombardy	16 July 2007	*	Faeces	MW984390	SAMN19667914
R1578	Offanengo (CR)	Lombardy	23 July 2007	R1572	Faeces	MW984403	SAMN19667927
R1579	Borgo San Giacomo (BS)	Lombardy	10 August 2007	*	Epithelium	MW984404	SAMN19667928
R1580	San Paolo (BS)	Lombardy	28 August 2007	*	Epithelium	MW984405	SAMN19667929
R1581	San Paolo (BS)	Lombardy	5 September 2007	*	Epithelium	MW984406	SAMN19667930
R1582	San Paolo (BS)	Lombardy	17 September 2007	*	Epithelium	MW984407	SAMN19667931
R1583	Orzinuovi (BS)	Lombardy	12 October 2007	*	Epithelium	MW984408	SAMN19667932
R1584	San Paolo (BS)	Lombardy	12 October 2007	*	Epithelium	MW984409	SAMN19667933
R1585	Orzinuovi (BS)	Lombardy	12 October 2007	*	Epithelium	MW984410	SAMN19667934
R1586	Borgo San Giacomo (BS)	Lombardy	22 October 2007	R1583	Epithelium	MW984411	SAMN19667935
R1587	Borgo San Giacomo (BS)	Lombardy	22 October 2007	R1583	Epithelium	MW984412	SAMN19667936

SRA = Short Read Archive. * Source farm with confirmed epidemiological link is known, but sequence of the corresponding virus is not available.

**Table 2:** SVDV amino acid residues evolving differently among the phases of the 2006–2007 SVD epidemic.

Region	Residue	α	β (Background)	β (First Phase)	β (Second Phase)	*p*	q	Pairwise Test
VP2	143	0	0	0	23.02	0.106	1	B
2A	999	0.073	0	0	15.123	0.101	1	BS
2C	1332	0	0	0	19.789	0.123	1	B
3D	2054	0	0	57.206	0	0.013	1	BF, FS

α = site-specific synonymous rate; β = site-specific nonsynonymous rate. The individual pairwise test was set as follows: BF = Background vs. First Phase, BS = Background vs. Second Phase, FS = First Phase vs. Second Phase. Background comprises branches of variants circulating in southern Italy. Permutation *p*-value was set at ≤0.1.

**Table 3:** SVDV amino acid residues estimated to be under directional selection in the 2006–2007 SVD epidemic. For each residue the observed residue composition, the root state, and target substitutions are reported.

Region	Residue	Composition	Root	Inferred Substitution		BF
2A	861	F_7_, V_73_	V	V 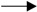 F_7_		1378.13
2C	1242	F_5_, I_75_	I	I 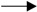 F_5_		360.36
3D	2054	A_21_, V_59_	V	A 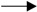 V_2_,	V 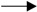 A_3_	5343.70

BF = Bayes Factor. Cut-off significance level was set at BF ≥ 100.

## Data Availability

The full-length SVDV genome sequences generated from this study have been deposited in GenBank with the following accession numbers: MW984370-MW984412 (Table 1). The raw NGS data have been deposited in the NCBI Short Read Archive with BioProject accession number PRJNA736881.

## Supplementary Materials

Supplementary materials can be found at https://www.mdpi.com/article/10.3390/v13071186/s1.
