# Supplementary material for: Retrospective Characterization of the 2006–2007 Swine Vesicular Disease Epidemic in Northern Italy by Whole Genome Sequence Analysis
Source: Viruses. 2021 Jun 22;13(7):1186. doi: 10.3390/v13071186 (PMC8310173; doi:10.3390/v13071186)
Supplement: Supplementary file 1 [file viruses-13-01186-s001.zip › viruses-1249678-supplementary.pdf]

Supplementary Figures

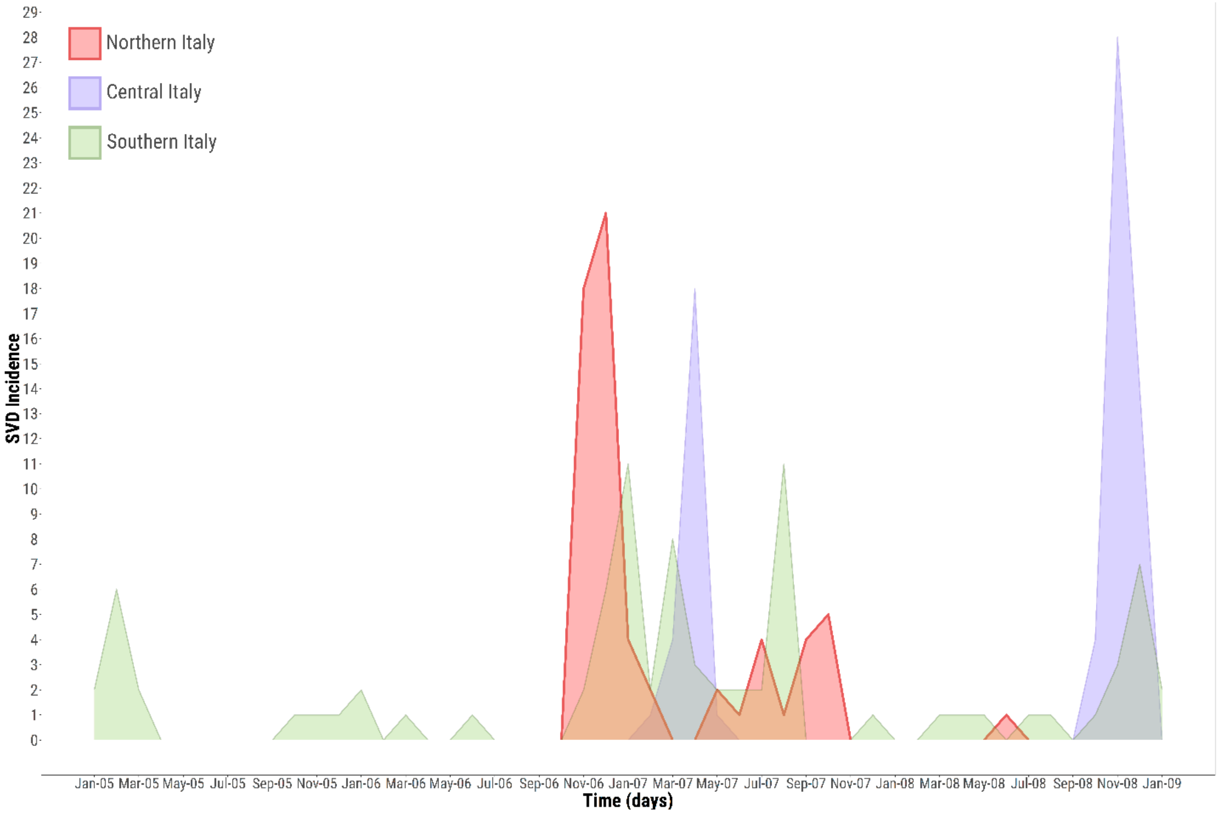

Figure S1. Epidemic curves reporting incidence estimates derived from the official reported cases of SVD in Northern, Central and Southern Italy between January 2005 and January 2009. The red epidemic curve of Northern Italy refers to the 2006-2007 SVD epidemic.

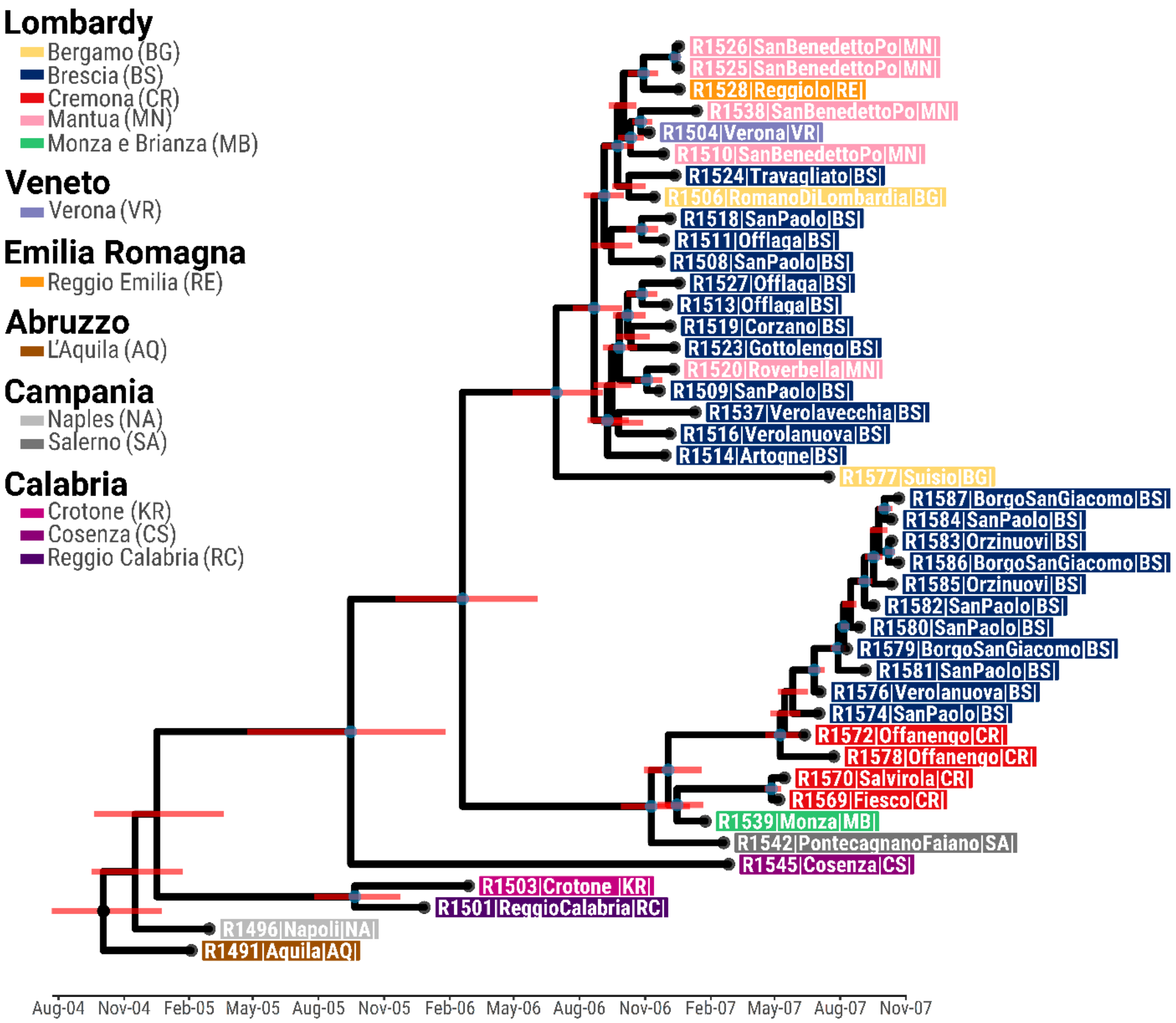

Figure S2. Time-calibrated Bayesian MCC tree inferred for the 2006-2007 SVD epidemic in Northern Italy using n = 43 whole-genome sequences. Nodes with a posterior support of >0.75 are identified in blue. Node bars describe the uncertainty region of the estimated date for each node (95% BCI).
